# Protease Nexin-1 in the Cardiovascular System: Wherefore Art Thou?

**DOI:** 10.3389/fcvm.2021.652852

**Published:** 2021-03-31

**Authors:** Celina Madjene, Alexandre Boutigny, Marie-Christine Bouton, Veronique Arocas, Benjamin Richard

**Affiliations:** ^1^LVTS, INSERM, U1148, Paris, France; ^2^Université de Paris, Paris, France; ^3^X. Bichat Hospital, Paris, France; ^4^Université Sorbonne Paris Nord, Villetaneuse, France

**Keywords:** PN-1, atherosclerosis, aneurysm, fibrosis, serpinE2, heart failure, smooth muscle cell

## Abstract

The balance between proteases and protease inhibitors plays a critical role in tissue remodeling during cardiovascular diseases. Different serine protease inhibitors termed serpins, which are expressed in the cardiovascular system, can exert a fine-tuned regulation of protease activities. Among them, protease nexin-1 (PN-1, encoded by *SERPINE2*) is a very powerful thrombin inhibitor and can also inactivate plasminogen activators and plasmin. Studies have shown that this serpin is expressed by all cell subpopulations in the vascular wall and by circulating cells but is barely detectable in plasma in the free form. PN-1 present in platelet granules and released upon activation has been shown to present strong antithrombotic and antifibrinolytic properties. PN-1 has a broad spectrum of action related to both hemostatic and blood vessel wall protease activities. Different studies showed that PN-1 is not only an important protector of vascular cells against protease activities but also a significant actor in the clearance of the complexes it forms with its targets. In this context, PN-1 overexpression has been observed in the pathophysiology of thoracic aortic aneurysms (TAA) and during the development of atherosclerosis in humans. Similarly, in the heart, PN-1 has been shown to be overexpressed in a mouse model of heart failure and to be involved in cardiac fibrosis. Overall, PN-1 appears to serve as a “hand brake” for protease activities during cardiovascular remodeling. This review will thus highlight the role of PN-1 in the cardiovascular system and deliver a comprehensive assessment of its position among serpins.

## Introduction

Protease Nexin-1 (PN-1) is a 50-kDa glycoprotein encoded by the *SERPINE2* gene on human chromosome 2 (1). Phylogenetically, it is the closest relative to plasminogen activator inhibitor type-1 (PAI-1 or serpinE1) (2). The serpins comprise a superfamily of proteins that share a conserved tertiary structure. Serpins include inhibitors of serine and papain-like cysteine proteases and non-inhibitory members with other biological functions. PN-1 is a cellular serpin found within diverse organs, such as brain, male and female reproductive systems, kidneys and lungs. PN-1 is also largely expressed in the vessels and the heart (3). This review thus aims to focus on its role in the pathophysiological responses of the cardiovascular system.

## Biochemical Properties of PN-1

PN-1 inhibits a broad range of serine proteases explaining its physiological role in various processes ranging from coagulation and fibrinolysis to tissue remodeling and inflammation. *In vitro* kinetic assays showed that PN-1 reacts rapidly with trypsin and thrombin, with an association rate constant (Ka) of ~2.10^6^ M^−1^.s^−1^ (4). The Ka values for the other target proteases including urokinase plasminogen activator (uPA) (4, 5), plasmin and factor XIa (6) are at least 10-fold lower, and 400-fold lower for tissue plasminogen activator (tPA) (4, 5) and activated protein C (7). PN-1 has also been shown to inhibit Factor VII-activating protease (FSAP) (8). As for many other serpins, PN-1 has a high affinity for heparin or heparan sulfate proteoglycans, which targets it to the pericellular space and strongly increases its ability to inhibit thrombin (9–11), thereby making this latter its preferred target. Indeed, unfractionated heparin is responsible for up to a 1,000-fold increase of the Ka value for thrombin, but only a ~10-fold increase for most other proteases and has no impact on the Ka value for plasmin. The crystal structure of the complex between thrombin, PN-1 and heparin demonstrated that heparin acts as a bridge between the serpin and the protease, leading to a ternary complex and enhancing the rate of complex formation (12). The protease-binding site (named the reactive center loop) of PN-1 is situated at the carboxy-terminal end of protein. The reactive site (P1–P′1) represented by the Arg346–Ser347 bond is cleaved by the target serine protease, which results in the formation of a covalent SDS- and heat-stable enzyme–PN-1 complex where both the protease and PN-1 become inactivated (10).

## PN-1 in the Vascular System

PN-1 does not circulate in plasma, but is present in blood cells, including platelets (13, 14) and monocytes (14). Active PN-1 is released from platelet α-granules during their activation. Platelet PN-1 displays anti-thrombotic properties *via* its ability to block thrombin generation and activity (15). This was illustrated by *in vivo* studies showing an important acceleration of the induction of thrombus formation after vascular injury in PN-1-deficient mice compared to wild-type mice (15). Platelet PN-1 also displays anti-fibrinolytic properties thanks to its ability to block plasmin generation and activity (16), as illustrated *in vivo* with PN-1-deficient mice that display accelerated and enhanced thrombolysis following treatment with tPA (16). Thus, both PAI-1 and PN-1 may play complementary roles in maintaining the fibrin clot, and therefore largely participate in the resistance of platelet-rich clots to thrombolysis.

The first report of the presence of PN-1 in the vasculature consisted of immunohistochemical studies demonstrating an abundance of PN-1 around cerebral blood vessels (17). Later, PN-1 expression was evidenced in the vascular wall where it is expressed by endothelial cells (18, 19), vascular smooth muscle cells (vSMCs) (20) and fibroblasts (21, 22). Importantly, it is retained at the cell surface of vascular cells and within the extracellular matrix (ECM) of the vessel wall due to its high affinity for heparin sulfate proteoglycans (22) and its ability to bind to the low-density lipoprotein receptor-related protein 1 (LRP1) of the scavenger receptor family (23, 24). PN-1 is expressed by endothelial cells and interacts with thrombomodulin, a high affinity thrombin ligand expressed on the endothelial cell membrane that plays an important role in the regulation of coagulation *via* the activation of the natural anticoagulant protein C. PN-1-thrombomodulin interaction favors the inhibition of fibrin formation and limits the generation of activated protein C and thrombin activatable fibrinolysis inhibitor (18). Endothelial PN-1 was also shown to protect the endothelial protein C receptor from endogenous shedding, thereby favoring the cytoprotective effects of activated protein C (25). Deficiency of PN-1 in mice does not generate a spontaneous vascular phenotype compromising their survival. However, endothelial PN-1 plays a role in physiological angiogenesis. Indeed, the retina from PN-1-deficient mice displayed increased vascularization with elevated capillary thickness and density, as well as an increased number of veins and arteries, compared to their wild-type littermates (26). Moreover, neovessel formation in Matrigel plug assays in PN-1-deficient mice, as well as the microvascular network sprouting from PN-1-deficient aortic rings, were both largely enhanced compared with their respective controls (27). These data clearly illustrate the important anti-angiogenic potential of vascular PN-1.

## PN-1 in Vascular Diseases

### PN-1 and Atherosclerosis

Atherosclerosis is a disease characterized by the thickening of the blood vessel wall due to the formation of plaques in the subendothelial intimal space. It involves endothelial cell dysfunction resulting in an alteration of endothelial permeability, allowing the penetration and accumulation of low-density lipoprotein (LDL) particles in the vessel wall where they are susceptible to oxidation. Monocytes are also implicated and transmigrate into the intima where they differentiate into macrophages, becoming foam cells after ingestion of oxidized LDL. VSMC proliferation and migration from the media to the intimal layer, as well as their phenotypic shift into foam cells, are also important features of atherosclerosis development. vSMCs present in the intimal layer form a fibrous cap that contains the plaque. The rupture of the fibrous cap leads to thrombus formation causing blockage of the blood flow (28).

An unbalanced ratio between proteases and their inhibitors is involved throughout the pathophysiology of atherosclerosis. Excessive thrombin, uPA/tPA or plasmin activities are indeed involved in the chronic evolution of the plaque. An important question thus concerns the regulation of these proteases in the vessel wall. In this context, serpins increasingly appear to be critical in regulating protease activity in arterial lesions. Among them, PN-1 has emerged as a key regulator in vascular biology even though its precise mechanism of action remains to be deciphered.

Immunohistochemical studies demonstrated the presence of PN-1 in the healthy vascular wall and particularly in vSMCs (20). PN-1 has also been shown to be associated with vSMCs in advanced carotid atherosclerotic lesions, but also with macrophages and platelets (14, 29). Accumulation of PN-1 was detectable in very early lesions and was increased in complicated plaques: globally, PN-1 was present in the cap, in the necrotic core and in the mural thrombus (14, 30). In fact, the biological activity of PN-1 appears to be involved in the different stages of atherosclerotic plaque progression. During the early stage, PN-1 may be involved in endothelial dysfunction. Indeed, at the endothelial level, PN-1 has been shown to interact with thrombomodulin, a glycoprotein that transforms thrombin from a pro- to an anticoagulant protein (18). Thrombomodulin interaction with PN-1 accentuates the ability of the latter to inhibit thrombin. In advanced atherosclerotic plaques, PN-1 is largely expressed by platelets and inflammatory cells including monocytes/macrophages. In agreement with this observation, PN-1 has been shown to be up-regulated in lipopolysaccharide-activated monocytes and degraded in macrophages (14). Because monocytes/macrophages are exposed to an inflammatory environment in atherothrombotic lesions, PN-1 overexpression may represent a cell defense reaction against proteases present in the atherosclerotic plaque. Indeed, vSMCs synthesize and secrete tPA that is able to drive the conversion of plasminogen into plasmin at the cell surface, leading to matrix degradation, cell detachment, and death (31). However, PN-1 is also overexpressed by vSMCs in the advanced plaque where it is able to form covalent complexes with plasmin (30). Both endocytic LRP-1 and PN-1 are highly expressed in human atheroma, making PN-1 a crucial actor in plasmin internalization by vSMCs, *via* LRP-1 (30). PN-1 has also been shown to form covalent complexes with FSAP (8), a circulating protease found in human atherosclerotic plaques and supposed to play a regulatory role in their progression and vulnerability (32). The fibrous cap plays a crucial role in the development of atherosclerosis because its thickness is tightly related to the vulnerability of atherosclerotic plaques. PN-1 may also influence the thickness of the fibrous cap, by acting on the migration of vSMCs. Indeed, overexpression of PN-1 by vSMCs has been shown to significantly reduce their adhesion, spreading and migration on vitronectin, an adhesive protein found in atherosclerotic plaques (33). This effect is related to the high affinity of PN-1 to vitronectin, shown by direct-binding *in vitro* assays (11). Moreover, PN-1 can limit thrombin-induced vSMC proliferation (20) and (i) prevents the pro-apoptotic effect of high thrombin concentrations (34), (ii) inhibits plasminogen activation in the peri-cellular environment, and (iii) prevents plasmin-induced cell detachment (34). Taken together, these data raise the possibility that PN-1 overexpression during atherosclerosis could significantly influence the stability of the plaque. At the most complicated stage of atherosclerosis, rupture of the plaque can trigger localized, often occlusive, thrombus formation. PN-1 can thus also accumulate within thrombi generated during atherothrombosis since platelets are a reservoir of this serpin. Via its ability to inhibit plasmin generation and activity within the thrombus, platelet PN-1 is assumed to contribute to thrombus stabilization and is therefore also a non-negligible contributor to thrombus resistance to lysis (16).

Given its ubiquitous expression in the atheromatous lesions and its inhibitory activity against numerous deleterious proteases present in the atheroma, PN-1 can undoubtedly regulate the characteristics of the atherosclerotic plaque at different stages of development.

### PN-1 and Aneurysms

Aortic aneurysms are also diseases characterized by intense remodeling due to an imbalance in favor of proteolytic degradation of the vascular wall ECM, leading to progressive dilation and eventually to rupture. Despite various possible etiologies, all thoracic aneurysms of the ascending aorta (TAA) share common pathophysiological features leading to structural deterioration of the aortic wall. VSMCs apoptosis and the degradation of collagen and elastic fibers are the two principal modifications occurring within the medial layer characterizing TAA. The relevance of the antiprotease activity of PN-1 expressed by vSMCs has been emphasized by its ability to regulate *in vitro* pericellular plasminogen activation (35) and therefore cell resistance to proteolytic aggression, as observed during atherosclerosis. In human biopsies, PN-1 expression was found to be increased in the medial layer of TAA compared with the aortic medial layer from healthy donors and the protein colocalized with vSMCs. Interestingly, cultured vSMCs from TAA continued to display an increased level of PN-1 mRNA expression compared with control vSMCs (36). This was found to be due to the permanent epigenetic activation of the smad2 pathway *in vivo* in the arterial wall of TAA, an activation which persisted in cultures of vSMCs of TAA origin. Hence, human cultured vSMCs from TAA had a limited capacity to convert plasminogen into plasmin, and were therefore protected against apoptosis-induced detachment after plasminogen or plasmin treatment (36). Indeed, PN-1 overexpression was shown to be associated with aneurysmal dilatation, whereas the absence of PN-1 overexpression was associated with aortic dissections (36). Together, these data show that overproduction of PN-1 by vSMCs *in vivo* during TAA development may participate in the increased ability of the cells to resist the proteolytic environment.

The clearance of PN-1/plasmin complexes has also been addressed specifically in the TAA context. PN-1, LRP-1 and plasmin were shown to colocalize in the media of human TAA where PN-1 amounts correlated with plasmin activity (37). The uptake of PN-1/plasmin complexes was shown to be partly mediated by LRP-1 in vSMCs. These results strongly suggest that PN-1 might play a protective role *in vivo* during TAA development, as discussed for atherosclerosis, but further experimental animal models are required to fully understand its impact on TAA pathophysiology.

In contrast to TAA, the role of PN-1 in abdominal aortic aneurysms (AAA) has not yet been addressed. Previous reports have shown that the enzymes of the fibrinolytic system are also involved in AAA progression (38, 39) and local overexpression of PAI-1 in the mouse was accordingly reported to prevent the development of the disease (40). The role of the plasminergic system remains nevertheless incompletely understood (41) and the study of PN-1 in this context could provide new insights into the understanding of how proteases and their counter-regulators participate in the evolution of AAA.

## PN-1 in Cardiac Fibrosis

Myocardial fibrosis is an important pathophysiological process defined as an excessive accumulation of matrix proteins and is a well-established morbi-mortality marker. It increases myocardial stiffness, alters systolic function and contributes to malignant arrhythmias (42).

PN-1 in the heart has received less attention although it has been reported to be present in mouse heart (43). Moreover, in rats a high overexpression of PN-1 was described in *in vivo* heart failure models (44). Li et al. were the first to assess the role of PN-1 in cardiac fibrosis (45). They showed that both cardiomyocytes and myocardial fibroblasts express PN-1, even though the level of PN-1 expression in the former was only half that in the latter. They also found, in an *in vivo* mouse model of cardiac fibrosis induced by transverse aortic constriction (TAC), that collagen deposition was increased after 4 weeks, associated with a slight increase in PN-1 expression in the heart (45). Moreover, they showed that pro-fibrotic mediators like angiotensin II and transforming growth factor-β (TGF-β) could induce, in myocardial fibroblasts, an increased expression of collagen associated with PN-1 overexpression, at both the messenger and protein levels. Such an up-regulation of PN-1 induced by TGF-β has also been observed *in vitro* in human pulmonary fibroblasts (46). Reciprocally, the knockdown of PN-1 appears to partially attenuate cardiac fibrosis (45). However, cardiac expression of PN-1 is only partially impaired and these data do not allow us to draw clear conclusions as to the role of PN-1 in cardiac injury.

PN-1 appears to be importantly involved in fibrotic processes. Interestingly, depending on the affected tissue, PN-1 displays either anti-fibrotic properties as described in pulmonary fibrosis (47) or in contrast, pro-fibrotic properties as described here in cardiac fibrosis or as reported in scleroderma, a disease also characterized by ECM accumulation in skin and visceral tissue (48). The link between PN-1 and cardiac fibrosis can also be mediated, at least in part, by its antiprotease inhibitor activity, in particular by its ability to inhibit thrombin and uPA. Indeed, the direct inhibition of thrombin with dabigatran was shown to attenuate cardiac fibrosis and improve global cardiac function in a TAC murine model (49). The importance of the uPA/plasmin/matrix-metalloproteinase (MMP) system in collagen degradation has been well-characterized (50). PAI-1, a serpin close to PN-1, has also been shown to exert pro-or anti-fibrotic effects in different organs. The inhibition by PAI-1 of uPA- and tPA-mediated conversion of plasminogen to plasmin was shown to decrease plasmin-mediated MMP activation, and consequently to increase matrix accumulation and fibrosis in different tissues including lung, liver and kidney (51). In contrast, in the heart, PAI-1 protects mice from hypertension-induced cardiac fibrosis (52). Indeed, although PAI-1 is upregulated by TGF-β in numerous cell types (53), in the myocardium, PAI-1 was shown to inhibit TGF-β production specifically in cardiomyocytes (51).

More detailed studies are required to decipher the role of PN-1 in cardiac fibrosis. Indeed, in pathological conditions, such as pressure overload models or myocardial infarction, inflammation plays an important role in adaptative and inadaptive responses, where monocytes and macrophages are key components of the inflammatory pathophysiology (54). Because PN-1 is expressed by inflammatory cells and has been shown to be closely related to the inflammatory reaction in lung fibrosis, we can hypothesize that PN-1 can also participate in cardiac inflammation and consequently, in cardiac fibrosis.

## Conclusions

**Figure 1:**
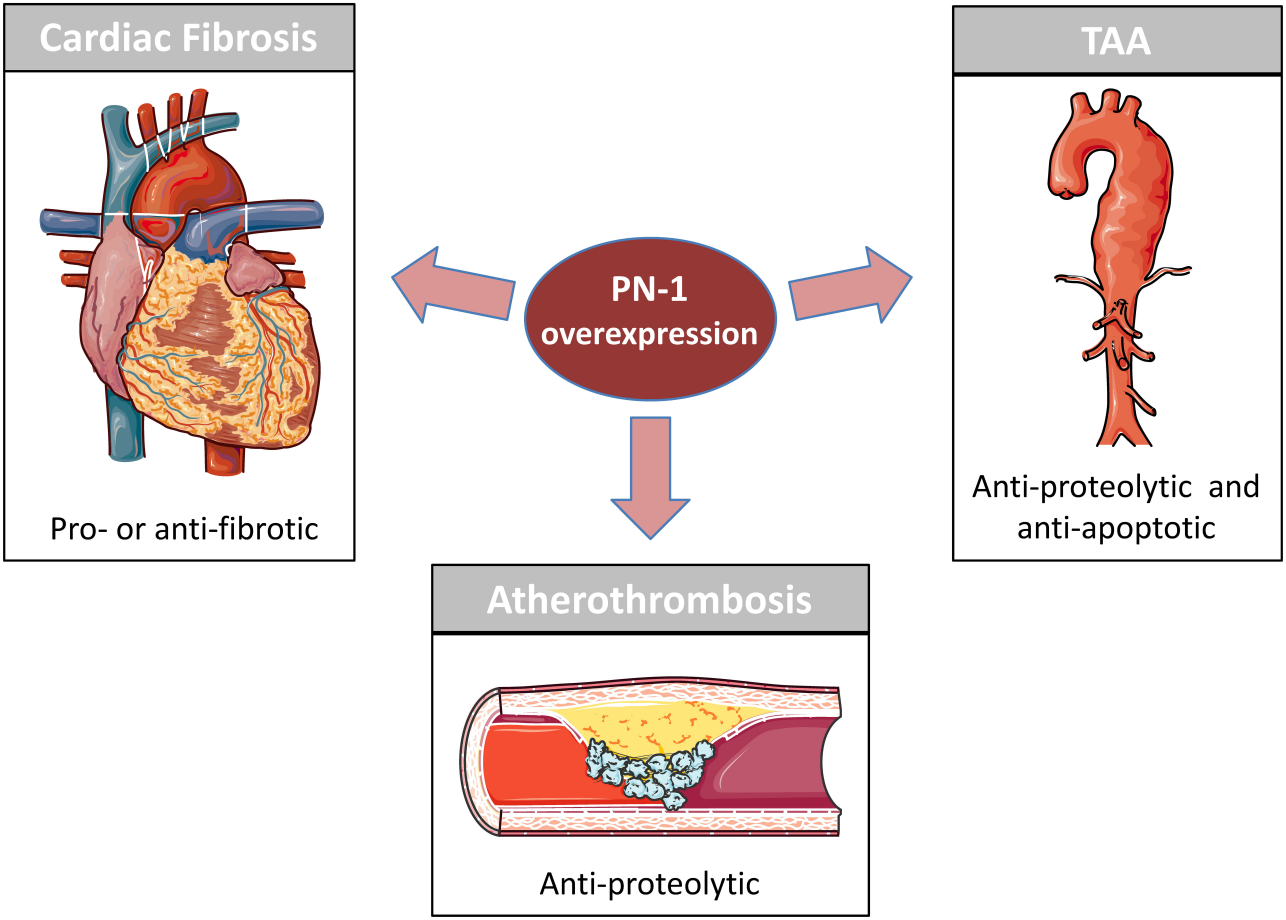
PN-1 in cardiovascular disease. PN-1 is overexpressed in Atherosclerosis, Cardiac Fibrosis and Thoracic Aortic Aneurysm (TAA). Previous studies have shown PN-1 to be an important protective actor in atherosclerosis and TAA by reducing the impact of the proteolytic environment on the vascular cells. PN-1 is also involved in cardiac fibrosis but can be either anti-fibrotic and protective or pro-fibrotic and deleterious depending on its targets (see Table 1).

The close relationships between PN-1 and proteases of the coagulation and fibrinolytic systems, as well as between PN-1 and the endocytic receptor LRP1, explain the impact of this serpin in the cardiovascular system (Table 1). Essentially, PN-1 participates in maintaining the homeostatic function of the arterial wall and the cardiac tissue, as illustrated by its overexpression in the different cardiovascular pathologies mentioned in this review (Figure 1).

**Table 1:** Expected effect of PN-1 in cardiovascular diseases depending on its targets or partners.

**Pathology**	**PN-1 targets or partners**	**Expected effect**	**References**
Thoracic and abdominal aortic aneurysms	Plasmin and LRP-1	Protective	(36, 37)
Atherosclerosis	Plasmin and LRP-1	Protective	(30)
Cardiac fibrosis	Thrombin	Protective	
	uPA, MMP, plasmin	Deleterious	(45)

## Author Contributions

CM and BR generated the figure and the table. VA and M-CB provided critical feedback and edited the review. All authors contributed to the review.

## Conflict of Interest

The authors declare that the research was conducted in the absence of any commercial or financial relationships that could be construed as a potential conflict of interest.
